# Greetings to the readers

**DOI:** 10.1080/0886022X.2019.1573499

**Published:** 2019-02-12

**Authors:** Tibor Fülöp

**Affiliations:** Department of Medicine, Division of Nephrology, Medical University of South Carolina, Charleston, SC, USA


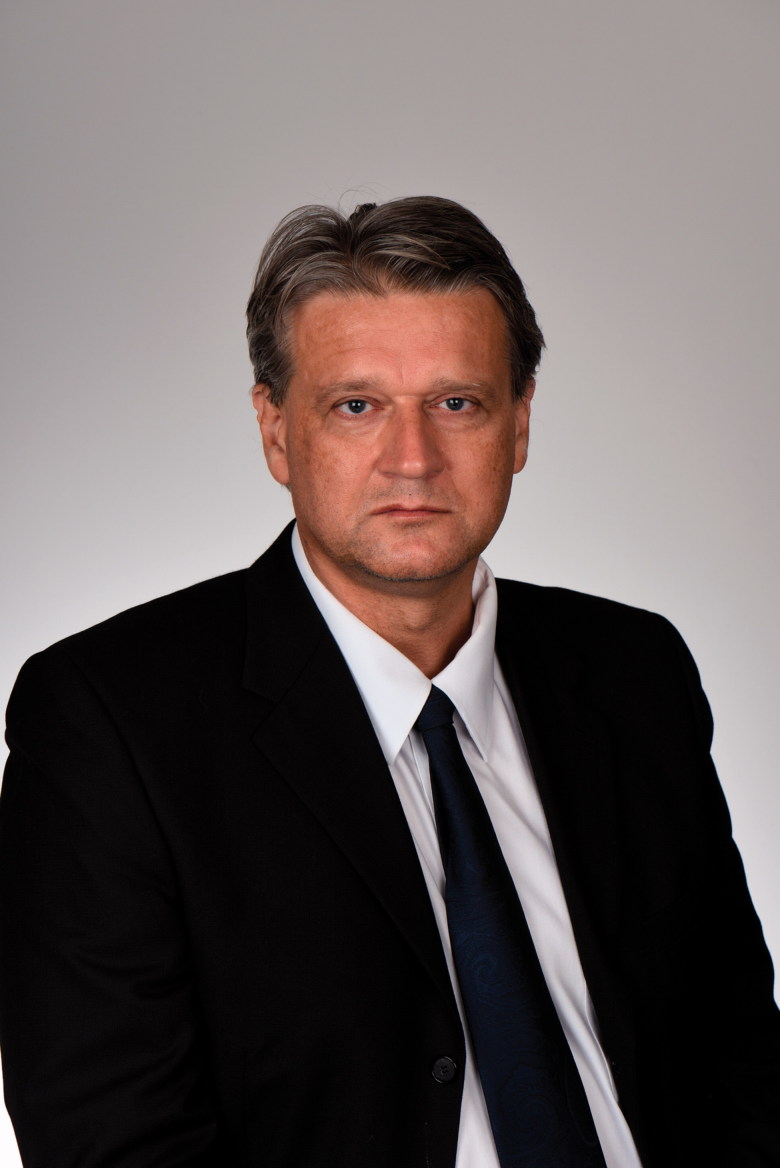


Dear Readers and Nephrology Colleagues,

As of January 1, 2019, I had the distinct privilege to assume the position of the new Editor-in-Chief for the journal *Renal Failure*. First of all, I would like to thank the previous Chief Editor, Dr. William F. Finn, for his years of dedicated work. Such transition will take place at a tumultuous time in academic publishing, but I hope to use this challenge as an opportunity to raise the Journal to the next level of academic exchange of information. Introducing myself, I am Board Certified Nephrologist and Internist and licensed to practice in both Mississippi and South Caroline (U.S.), as well as in the European Union (Hungary). Currently, I am a Full Professor of Medicine at the Medical University of South Carolina (MUSC) and Attending Physician for the MUSC’s Department of Medicine, Division of Nephrology and Ralph H. Johnson VA Medical Center and serving as Medical Director for Dialysis Units in the community and at the VA Medical Center. Earlier in my carrier, I have served on the faculty of several prestigious institutions, including the University of Mississippi, the University of Iowa, the University of Debrecen and the Semmelweis University in Hungary.

Over the last 2 years our Publisher, Taylor & Francis Online, and the dedicated personnel of the Editorial Office, have overseen the transition to an Open Access (OA) model of publishing and a dramatic increase of downloads for the *Journal* worldwide with an improved overall academic impact. OA publishing, while initially viewed with disdain by some of us, is now embracing ‘mainstream’ publications, increasingly becoming the norm of academic publishing. The success of journals such as *PLoS One* and *Scientific Reports* bears testimony to this transition. An increasing number of authors are now encouraged or required to publish OA by their funding bodies, institutions, or their employers. To that end, our Publisher will also offer significant support for authors from countries with lesser financial means: corresponding authors with primary affiliations in countries defined by the World Bank as ‘Low-Income Economies’ can apply for a 100% article processing change (APC) waiver; those from ‘Lower-Middle Income Economies’ can apply for a 50% APC discount. Additionally, corresponding authors with primary affiliations based in one of the EIFL (Electronic Information for Libraries) network countries may be eligible for a 100% or 50% discount as well.

Over the last couple of years, we have seen the *Journal* publish highly impactful papers on a wealth of issues, including meta-analyses on the link between proton-pump inhibitors and hypomagnesemia [1], on the impact of overhydrated status and low lean tissue in end-stage renal disease (ESRD) patients [2], on uric acid lowering therapy and chronic kidney disease progression [3] and a systemic review and meta-analysis further confirming cinacalcet’s lack of impact on survival in (ESRD) patients [4]. Single-center studies published in the *Journal* have explored the impact of AKI in donors on renal graft survival subsequent to renal transplantation [5], the risk of AKI after transcatheter aortic valve placement [6] and quality of life, as well as the correlation of personality profiles and coping styles with clinical outcomes in ESRD patients on maintenance hemodialysis [7]. Additional publications in the *Journal* have also explored the frequency and clinical characteristics of invasive fungal infections in renal transplant recipients [8], the value of combination biomarkers in predicting renal impairment after a cardiovascular bypass procedure [9], the clinical characteristics of sepsis-induced AKI from China [10], post-partum renal injury from India [11] outcomes and characteristics of AKI in hospitalized patients from sub-Saharan Africa. Emerging technologies, such as bioimpedance spectroscopy-assisted volume status assessment also received prominent attention in the *Journal* over this period [2,12]. Along with human studies, the *Journal* has published important works on animal models of AKI as well. These have included work on the potential protective role of N-acetylcysteine in oxalate-induced AKI in a rat model [13], the protective effect of berberine against gentamycin-induced nephrotoxicity in rats [14] and its possible underlying mechanisms, and explored the protective role of heme oxygenase-1 on the course of cisplatin-induced nephrotoxicity [15].

Nephrology in the acute care setting is moving into an era of cooperation and interaction between several disciplines to address the complex needs stemming from the patients’ multi-faceted problems. We should remember that in most settings, renal replacement therapy in the Intensive Care Units is still associated with at least 50% in mortality rate [10,16–18] – obviously, the current status quo of practice (and knowledge) is insufficient and the one thing that cannot be justified is inertia. To mention just a few examples, there is exciting literature emerging on fluid overload and volume determination in critically ill patients with acute kidney injury (AKI) [19–22], the impact of other organ failures (liver, heart) in AKI [23], on peritoneal dialysis as a viable alternative for continuous renal replacement therapy [24,25] and on the uniqueness of medication dosing in those with critical illness receiving renal replacement therapy [26,27]. All these issues will likely expand in the future and attract a global readership.

To stay relevant in the globally connected world, it is important to have a global representation and reverberations of our publications. In my role as Editor-in-Chief, I will look forward to identifying talented and motivated young clinician-scientists to assist us with the onerous work of peer reviews and to seeing them on our Editorial Board (EB) as the Journal grows. We will strongly seek to develop a pool of peer reviewers and EB members who are reflecting, in composition and interest, the Journal’s international readership. As an added benefit, our publication fee is already substantially reduced for the EB members. We also sincerely hope the next generation of young clinician-scientists will find it worthy not only to read the Journal but also to publish with us. We look forward to further attracting a vibrant and international readership who are likely to return to the journal’s website after their initial positive experiences. There is great competition going on in the modern world to attract quality papers and readers. Those who read papers are the most likely to organize their own studies and publish new ones. We will strongly consider the publication of novel ideas if the underlying science is sound, even if certain questions might remain unanswered in the study.

Our impact factor is currently standing at 1.44 and we look forward to attracting exciting papers on a wealth of issues but focus predominantly on AKI and aspects of critical care nephrology [17,28,29]. None of us went into Medicine hoping to find a boring job, and academic writing – so it seems to so many of us – is akin to a child’s birth: it is immensely joyous and exhilarating to bring something into this world, which never existed before. The future is ours to make it better and I hope to find fellow travelers on this road. Let’s make it happen!

## References

[1] Cheungpasitporn W, Thongprayoon C, Kittanamongkolchai W, Srivali N, Edmonds PJ, Ungprasert P, et al. Proton pump inhibitors linked to hypomagnesemia: a systematic review and meta-analysis of observational studies. Ren Fail. 2015;37(7):1237–1241. 26108134 10.3109/0886022X.2015.1057800

[2] Hwang SD, Lee JH, Lee SW, Kim JK, Kim M-J, Song JH Risk of overhydration and low lean tissue index as measured using a body composition monitor in patients on hemodialysis: a systemic review and meta-analysis. Ren Fail. 2018;40(1):51–59. 29347876 10.1080/0886022X.2017.1419963 PMC6014525

[3] Liu X, Zhai T, Ma R, Luo C, Wang H, Liu L Effects of uric acid-lowering therapy on the progression of chronic kidney disease: a systematic review and meta-analysis. Ren Fail. 2018;40(1):289–297. 29619870 10.1080/0886022X.2018.1456463 PMC6014338

[4] Sekercioglu N, Busse JW, Sekercioglu MF, Agarwal A, Shaikh S, Lopes LC, et al. Cinacalcet versus standard treatment for chronic kidney disease: a systematic review and meta-analysis. Ren Fail. 2016;38(6):857–874. 27137817 10.3109/0886022X.2016.1172468

[5] Zheng YT, Chen CB, Yuan XP, Wang CX Impact of acute kidney injury in donors on renal graft survival: a systematic review and Meta-Analysis. Ren Fail. 2018;40(1):649–656. 30396304 10.1080/0886022X.2018.1535982 PMC6225519

[6] Thongprayoon C, Cheungpasitporn W, Srivali N, Harrison AM, Kittanamongkolchai W, Greason KL, et al. Transapical versus transfemoral approach and risk of acute kidney injury following transcatheter aortic valve replacement: a propensity-adjusted analysis. Ren Fail. 2017;39(1):13–18. 27767371 10.1080/0886022X.2016.1244072 PMC6014512

[7] D’Onofrio G, Simeoni M, Rizza P, Caroleo M, Capria M, Mazzitello G, et al. Quality of life, clinical outcome, personality and coping in chronic hemodialysis patients. Ren Fail. 2017;39(1):45–53. 27778533 10.1080/0886022X.2016.1244077 PMC6014518

[8] Patel MH, Patel RD, Vanikar AV, Kanodia KV, Suthar KS, Nigam LK, et al. Invasive fungal infections in renal transplant patients: a single center study. Ren Fail. 2017;39(1):294–298. 28085530 10.1080/0886022X.2016.1268537 PMC6014505

[9] Prowle JR, Calzavacca P, Licari E, Ligabo EV, Echeverri JE, Bagshaw SM, et al. Combination of biomarkers for diagnosis of acute kidney injury after cardiopulmonary bypass. Ren Fail. 2015;37(3):408–416. 25585949 10.3109/0886022X.2014.1001303 PMC4544762

[10] Shum H-P, Kong HH-Y, Chan K-C, Yan W-W, Chan TM Septic acute kidney injury in critically ill patients–a single-center study on its incidence, clinical characteristics, and outcome predictors. Ren Fail. 2016;38(5):706–716. 26981621 10.3109/0886022X.2016.1157749

[11] Eswarappa M, Madhyastha PR, Puri S, Varma V, Bhandari A, Chennabassappa G Postpartum acute kidney injury: a review of 99 cases. Ren Fail. 2016;38(6):889–893. 27319810 10.3109/0886022X.2016.1164015

[12] Rymarz A, Gibińska J, Zajbt M, Piechota W, Niemczyk S Low lean tissue mass can be a predictor of one-year survival in hemodialysis patients. Ren Fail. 2018;40(1):231–237. 29620449 10.1080/0886022X.2018.1456451 PMC6014293

[13] Shimizu MH, Gois PH, Volpini RA, Canale D, Luchi WM, Froeder L, et al. N-acetylcysteine protects against star fruit-induced acute kidney injury. Ren Fail. 2017;39(1):193–202. 27845599 10.1080/0886022X.2016.1256315 PMC6014349

[14] Adil M, Kandhare AD, Dalvi G, Ghosh P, Venkata S, Raygude KS, et al. Ameliorative effect of berberine against gentamicin-induced nephrotoxicity in rats via attenuation of oxidative stress, inflammation, apoptosis and mitochondrial dysfunction. Ren Fail. 2016;38(6):996–1006. 27056079 10.3109/0886022X.2016.1165120

[15] Behiry S, Rabie A, Kora M, Ismail W, Sabry D, Zahran A Effect of combination sildenafil and gemfibrozil on cisplatin-induced nephrotoxicity; role of heme oxygenase-1. Ren Fail. 2018;40(1):371–378. 29707997 10.1080/0886022X.2018.1455596 PMC6014327

[16] Brar H, Olivier J, Lebrun C, Gabbard W, Fulop T, Schmidt D Predictors of mortality in a cohort of intensive care unit patients with acute renal failure receiving continuous renal replacement therapy. Am J Med Sci. 2008;335(5):342–347. 18480649 10.1097/MAJ.0b013e3181571f56

[17] Wu L, Zhang P, Yang Y, Jiang H, He Y, Xu C, et al. Long-term renal and overall survival of critically ill patients with acute renal injury who received continuous renal replacement therapy. Ren Fail. 2017;39(1):736–744. 29199512 10.1080/0886022X.2017.1398667 PMC6446161

[18] Cho AY, Yoon HJ, Lee KY, Sun IO Clinical characteristics of sepsis-induced acute kidney injury in patients undergoing continuous renal replacement therapy. Renal failure. 2018;40(1):403–409. 30015549 10.1080/0886022X.2018.1489288 PMC6052425

[19] Fülöp T, Pathak MB, Schmidt DW, Lengvárszky Z, Juncos JP, Lebrun CJ, et al. Volume-related weight gain and subsequent mortality in acute renal failure patients treated with continuous renal replacement therapy. ASAIO J. 2010;56(4):333. 20559136 10.1097/MAT.0b013e3181de35e4 PMC2895683

[20] Chen H, Wu B, Gong D, Liu Z Fluid overload at start of continuous renal replacement therapy is associated with poorer clinical condition and outcome: a prospective observational study on the combined use of bioimpedance vector analysis and serum N-terminal pro-B-type natriuretic peptide measurement. Crit Care. 2015;19(1):135. 25879573 10.1186/s13054-015-0871-3 PMC4391528

[21] Vaara ST, Korhonen A-M, Kaukonen K-M, Nisula S, Inkinen O, Hoppu S, et al. Fluid overload is associated with an increased risk for 90-day mortality in critically ill patients with renal replacement therapy: data from the prospective FINNAKI study. Crit Care. 2012;16(5):R197. 23075459 10.1186/cc11682 PMC3682299

[22] Garzotto F, Ostermann M, Martin-Langerwerf D, Sanchez-Sanchez M, Teng J, Robert R, et al. The Dose Response Multicentre Investigation on Fluid Assessment (DoReMIFA) in critically ill patients. Crit Care. 2016;20(1):196. 27334608 10.1186/s13054-016-1355-9 PMC4918119

[23] Ponce D, Goes C, Oliveira M, Balbi A Peritoneal Dialysis for the Treatment of Cardiorenal Syndrome Type 1: A Prospective Brazilian Study. Perit Dial Int. 2017;37(5):578–583. 28931700 10.3747/pdi.2016.00217

[24] Ponce D, Buffarah MB, Goes C, Balbi A Peritoneal Dialysis in Acute Kidney Injury: Trends in the Outcome across Time Periods. PLoS One. 2015;10(5):e0126436. 25965868 10.1371/journal.pone.0126436 PMC4428622

[25] Fülöp T, Zsom L, Tapolyai MB, Molnar MZ, Rosivall L Volume-related weight gain as an independent indication for renal replacement therapy in the intensive care units. J Renal Inj Prev. 2017;6(1):35–42. 28487870 10.15171/jrip.2017.07 PMC5414517

[26] Ponce D, Zamoner W, Freitas FM, Balbi A, Awdishu L Vancomycin Removal During High-Volume Peritoneal Dialysis in Acute Kidney Injury Patients: A Prospective Cohort Clinical Study. Kidney Int Rep. 2019;4(1):112–118. 10.1016/j.ekir.2018.09.014 PMC6308823 30596174

[27] Eyler RF, Mueller BA, Medscape Antibiotic dosing in critically ill patients with acute kidney injury. Nat Rev Nephrol. 2011;7(4):226–235. 21343897 10.1038/nrneph.2011.12

[28] Cho AY, Yoon HJ, Lee KY, Sun IO Clinical characteristics of sepsis-induced acute kidney injury in patients undergoing continuous renal replacement therapy. Ren Fail. 2018;40(1):403–409. 30015549 10.1080/0886022X.2018.1489288 PMC6052425

[29] Gameiro J, Goncalves M, Pereira M, Rodrigues N, Godinho I, Neves M, et al. Obesity, acute kidney injury and mortality in patients with sepsis: a cohort analysis. Ren Fail. 2018;40(1):120–126. 29388454 10.1080/0886022X.2018.1430588 PMC6014496

